# Item-Level Scores on the Boston Naming Test as an Independent Predictor of Perirhinal Volume in Individuals with Mild Cognitive Impairment

**DOI:** 10.3390/brainsci13050806

**Published:** 2023-05-16

**Authors:** Matteo De Marco, Martina Bocchetta, Annalena Venneri, for the Alzheimer’s Disease Neuroimaging Initiative

**Affiliations:** 1 Centre for Cognitive and Clinical Neuroscience, Division of Psychology, Department of Life Sciences, College of Health, Medicine and Life Sciences, Brunel University London, London UB8 3PH, UK; matteo.demarco@brunel.ac.uk (M.D.M.); martina.bocchetta@brunel.ac.uk (M.B.); 2 Dementia Research Centre, Department of Neurodegenerative Disease, UCL Queen Square Institute of Neurology, University College London, London WC1E 6BT, UK; 3 Department of Medicine and Surgery, University of Parma, 43125 Parma, Italy

**Keywords:** body–object interaction, Alzheimer’s disease, semantic memory, confrontational naming, lexical abilities, MRI, neuroimaging

## Abstract

We explored the methodological value of an item-level scoring procedure applied to the Boston Naming Test (BNT), and the extent to which this scoring approach predicts grey matter (GM) variability in regions that sustain semantic memory. Twenty-seven BNT items administered as part of the Alzheimer’s Disease Neuroimaging Initiative were scored according to their “sensorimotor interaction” (SMI) value. Quantitative scores (i.e., the count of correctly named items) and qualitative scores (i.e., the average of SMI scores for correctly named items) were used as independent predictors of neuroanatomical GM maps in two sub-cohorts of 197 healthy adults and 350 mild cognitive impairment (MCI) participants. Quantitative scores predicted clusters of temporal and mediotemporal GM in both sub-cohorts. After accounting for quantitative scores, the qualitative scores predicted mediotemporal GM clusters in the MCI sub-cohort; clusters extended to the anterior parahippocampal gyrus and encompassed the perirhinal cortex. This was confirmed by a significant yet modest association between qualitative scores and region-of-interest-informed perirhinal volumes extracted *post hoc*. Item-level scoring of BNT performance provides complementary information to standard quantitative scores. The concurrent use of quantitative and qualitative scores may help profile lexical–semantic access more precisely, and might help detect changes in semantic memory that are typical of early-stage Alzheimer’s disease.

## 1. Introduction

Semantic memory (SM) is a multidimensional ability that enables the processing of semantic knowledge (SK). SK representations are stored throughout the entire cortex [1,2], and, as with information processed by any other type of memory, these are learnt and, subsequently, accessed via mechanisms of encoding and retrieval, respectively. A very large meta-analysis based on an “Activation Likelihood Estimate” methodology indicates that the system responsible for encoding and retrieving SK is supported by a widespread cerebral network (the SM network) that involves the limbic and hippocampal/parahippocampal regions, but also extends to the inferoparietal, middle-temporal, and prefrontal cortices [3]. Additional evidence indicates that, aside from cerebral areas, the cerebellum is also involved in SM [4].

The processes of encoding and retrieval prompted by a SM task, however, typically also entail a certain number of executive demands. This is known as “semantic control” and identifies the resources that are deployed to manipulate SK, i.e., to select and elaborate task-related and context-related aspects (or nuances) of SK that are relevant for goal-oriented purposes [5]. A multidimensional body of findings (including neuroimaging and neuromodulatory evidence) indicates that semantic control is sustained by the prefrontal, temporal, and parietal portion of the aforementioned SM network [5,6]. However, the role of the parietal lobe in sustaining semantic control is not supported by meta-analytical findings [7]. Nonetheless, complementary evidence suggests that the posterior parietal cortex may serve a different, yet equally important function, by providing attentional resources to feature attendance [8], which, in the computational cascade of SM performance, precede the controlled processes of feature selection.

When SM is assessed in neurological settings, it is typically tested via retrograde memory tests. As a result, the measures emerging from these instruments depend on retrieval, not encoding abilities. Although the wider network sustaining semantic SM (inclusive of semantic control) is widespread across multiple brain regions, the core of retrieval mechanisms is linked to the mediotemporal lobe [9]. Whereas episodes (encoded and retrieved by episodic memory processes) are *context-rich* (i.e., are typically accompanied by temporal, spatial, emotional, or other situational contexts), SK is “computationally lighter” as it is *context-free* [10]. Data from adolescents and young adults with selective bilateral damage to the hippocampus acquired during development show preserved SM [11]. This indicates that the anterior parahippocampal region adjacent to the hippocampus (corresponding to the perirhinal cortex) is sufficient to process context-free SM [10]. Regional specialisation of the perirhinal cortex for SM retrieval has been supported by diverse sources of evidence, including data from healthy controls, as well as individuals with neurodegenerative decline, and non-human primates [12,13,14].

The link between SM retrieval and the integrity of the perirhinal cortex is particularly important for the study of early-stage Alzheimer’s disease (AD), as this is the earliest cortical region that shows neurofibrillary pathology [15]. Studies that followed up cohorts of participants who were healthy at baseline indicate that SM decline is the earliest measurable change in individuals who later develop AD [16,17,18]. Although indirectly, this association has also been supported by studies that have analysed the neuroanatomical properties of the sub-hippocampal cortex. Perirhinal thinning is observed in individuals with mild cognitive impairment (MCI) [19], and volumetric reductions are seen at the preclinical stage of AD in the entorhinal region adjacent to the perirhinal cortex [20].

Although this body of evidence provides a rationale for relying on SM tests to detect very early AD-related alterations [21,22], it is important to remember that SM performance is also supported by semantic control. This implies that the variability of standard SM test scores is influenced by abilities sustained by regions other than the perirhinal cortex. This is the case for the Category Fluency Test, for instance, which is a SM test, but it is also sustained by a considerable number of executive resources [23,24,25].

To overcome this limitation and expand the applicability of the Category Fluency Test, an alternative scoring approach has been designed. These are known as “item-level” scoring methods. Whereas traditional scoring procedures consist of a count of the words generated during test performance (a quantitative metric), item-level methods assign a difficulty score to individual words (a qualitative property of each word), based on the principle that, if SM is better preserved, a person is able to name more difficult words [26]. Item-level methods offer the opportunity to expand and enrich SM test profiles, based on the assumption that qualitative scores are less influenced by semantic control, and can thus represent a more genuine proxy of perirhinal integrity.

Item-level methods, however, have been mostly applied to the scoring of the Category Fluency Test, e.g., [27,28,29,30,31]. No study has yet investigated the added value of item-level scoring approaches applied to other SM tests. This study focuses on the Boston Naming Test (BNT), a word-retrieval test in which the participant is requested to name a set of black-and-white line drawings of various levels of semantic difficulty [32]. To investigate the added value of item-level scoring of the BNT, “sensorimotor interaction” (SMI; also known as “body–object interaction”) was selected as the item-level feature of interest. Although a range of dimensions exists, along which the semantic complexity/difficulty of words can be operationalised, such as category-dependent typicality [30], frequency of use [29], or age of acquisition [27,28], SMI was selected because it embeds aspects of neural processing that are linked to regions involved in somatosensory and motor processing [33], and that could thus be associated with distinct topological maps.

The following two hypotheses were formulated: (1) the item-level complexity of named words accounts for a significant portion of grey matter (GM) density, independently of that accounted for by quantitative BNT performance, and (2) this independent portion of variability extends to associative regions that are involved in somatosensory and motor processing.

### BNT Literature Review

The BNT is a test of picture naming that, over the years, has been used in clinical research to characterise anomias and deficits of semantic processing in individuals with neurological conditions. Individuals at a mild stage of dementia of the AD type perform worse than controls on the BNT [34], and their performance is a significant predictor of further cognitive decline after 30 months [35]. Individuals with dementia of the AD type also perform significantly worse on the BNT than individuals with a form of dementia with Lewy Bodies of comparable severity [36], and the performance of individuals with a diagnosis of frontotemporal dementia depends on the pathophysiological profile of the disease. Individuals with a behavioural variant obtain similar levels of performance to that of individuals with AD dementia, whereas individuals with a semantic variant perform significantly worse [37,38]. Data indicate that the total BNT score of individuals with amnestic MCI is within the range of normality. These individuals, however, tend to make disproportionately more semantic errors (such as paraphasias and circumlocutions) [39], but, at the same time, they also tend to provide significantly fewer spontaneous responses than those of healthy controls, suggesting that naming difficulties in this group cannot be fully accounted for by SK disruption [40]. Findings obtained from neuroimaging studies provide further evidence in support of a range of diverse mechanisms characterising BNT performance in individuals with MCI and AD dementia. Reduced BNT performance at the MCI stage is associated with GM density in the left anterior temporal lobe. This association extends to the bilateral mediotemporal lobe when individuals with MCI and dementia of the AD type are analysed as part of a single inferential model [41], indicating that disease processes may contribute to altering the underlying neurocognitive mechanisms of BNT performance at some point along the clinical trajectory. Moreover, changes in volume in the left hippocampus in a sample defined along the continuum between MCI and AD dementia is predictive of longitudinal BNT decline over a span of ~2 years [42]. When MCI individuals are evaluated as a function of their subsequent progression to AD dementia, performance on the BNT appears to be a significant predictor of atrophy progression. AD converters who were low-BNT MCI performers, in fact, develop significantly more atrophy (than that of high-BNT MCI performers) in a large set of prefrontal, temporal, and parietal regions one year after conversion to dementia [43]. Taken together, these findings suggest that the neurological resources that support performance on the BNT at the MCI stage are influenced by disease mechanisms, but are also associated with the integrity of a series of regions known to sustain memory retrieval and semantic control.

In light of this heterogeneous pattern, the aforementioned study hypotheses were addressed to test the added value of a BNT item-level score, under the assumption that this qualitative index predicts patterns of GM density independent of those predicted by “standard” quantitative scores.

## 2. Methods

### 2.1. Participants

The Alzheimer’s Disease Neuroimaging Initiative (ADNI) database (adni.loni.usc.edu) was identified as an appropriate source of data to address the study hypotheses. The ADNI was launched in 2003 as a public–private partnership, led by Principal Investigator Michael W. Weiner, MD. The primary goal of ADNI has been to test whether serial magnetic resonance imaging (MRI), positron emission tomography, other biological markers, and clinical and neuropsychological assessment can be combined to measure the progression of MCI and early AD. For up-to-date information, see www.adni-info.org.

The online repository of the ADNI initiative includes item-level scores of individual BNT performances (see Section 2.2.1. for more details). These can be found within the “*Item Level Data*” database, a spreadsheet that includes scores from individual items of a number of tests and batteries [44]. As of April 2023, availability of item-level data is limited to the first phase of ADNI (i.e., “ADNI1”) that includes > 700 participants with a clinical diagnosis of “control”, “MCI”, or “AD dementia”. For the purpose of this study, control (*n* = 205) and MCI (*n* = 369) individuals with available cognitive and good quality neuroimaging data were considered for inclusion. As repeated BNT performance is characterised by practice effects [45], baseline data only were scrutinised to address the study hypotheses. All participants included in this study were between the ages of 55 and 90, had a Mini Mental State Examination score between 24 and 30, were fluent in English or Spanish, and were supported by a reliable informant that could contribute to the diagnostic procedures. All healthy controls had a Clinical Dementia Rating score of 0 and no symptoms indicating the presence of depression or cognitive impairment. MCI participants instead had a Clinical Dementia Rating score of 0.5, a subjective memory complaint, objective memory decline (as informed by education-corrected scores on Wechsler Memory Scale Logical Memory II), no decline in activities of daily living, and no dementia.

### 2.2. Materials

#### 2.2.1. Boston Naming Test Scores

Based on the original 60-item version of the test [32], a short version of the BNT is included as part of the ADNI neuropsychological battery, and this consists of the 30 odd (i.e., 1, 3, 5, 7, 9, etc.) trials only. Each individual BNT performance was initially inspected for data-cleaning purposes. At this stage, 8 control and 17 MCI datasets were discarded because of missing data on one or more BNT item responses (marked by ADNI as “999”), leaving a cohort of 197 cognitively normal controls and 352 individuals with MCI. Items were then classified as “correct” (if correctly named on the first attempt) or “incorrect” (including those named only after the provision of a cue).

The values for word-related SMI were obtained from a normative study carried out on a cohort of >1200 native English speakers [46]. SMI values were not available for 3 of the 30 words (“OCTOPUS”, “VOLCANO”, and “TRELLIS”). As a result, item-level scoring was carried out on the remaining 27 items. For each individual, the following scores were calculated: (1) a quantitative BNT score based on the 27 items that had a valid SMI value; and (2) a qualitative BNT score obtained by averaging item-level SMI scores of correctly named items. A qualitative BNT score could not be calculated for 2 MCI participants due to missing data, as they had correctly named <2 items. These datasets were discarded, and this left us with a sub-cohort of 350 MCI individuals. All participants included in this study were tested in English.

#### 2.2.2. MRI Processing

Volumetric T1-weighted images (all acquired at 1.5 T) were extracted from each individual MRI protocol archived by ADNI. This was carried out by minimising the temporal distance (in days) between BNT and MRI dates. Images were acquired in compliance with the ADNI1 MRI specification protocol [47]. All files were preprocessed and analysed with Statistical Parametric Mapping 12 (Wellcome Centre for Human Neuroimaging, London, UK), running under MATLAB (version R2014b; Mathworks Inc., Natick, MA, USA). Images were manually reoriented according to their bicommissural axis, and then they underwent segmentation into three tissue-specific probabilistic maps: GM, white matter, and cerebrospinal fluid. Global volumetric indices (in ml) were calculated for each native-space output file using the “get_totals” script (www0.cs.ucl.ac.uk/staff/g.ridgway/vbm/get_totals.m). The values of the three tissue maps were summed up for each participant to obtain individual indices of intracranial volumes. A brain–parenchymal ratio was then calculated (GM plus white matter volume divided by intracranial volume) to obtain proxies of tissue density. Finally, segmented GM maps were modulated, normalised to the Montreal Neurological Institute space, and smoothed (with a 6 mm full-width at half maximum Gaussian kernel) for the purpose of data modelling.

As the aim of this study was to establish the link between GM and both quantitative and qualitative aspects of BNT performance, an additional neuroanatomical index was computed. This was to regress out an aspect of variability that is typically associated with neurodegenerative changes due to AD, and that can constitute a major confound in this type of analysis. The WFU Pickatlas toolbox [48], in tandem with the human Brodmann Atlas, was used to define a left hippocampal region of interest (ROI). The “get_totals” script was then used to extract individual left hippocampal volumes from the set of GM tissue maps. To confirm the validity of these scores, maps of structural covariance were calculated (see Section 2.3 and Section 3 for more details).

### 2.3. Data Analyses

Two sets of multiple-regression inferential models were designed to test the linear association between GM maps and the quantitative and qualitative aspects of BNT performance. This was carried out separately for the sub-cohorts of controls and MCI individuals.

Model 1 tested the statistical effect of quantitative scoring (i.e., the count of correctly named items, out of 27). Four covariates were added to regress out aspects of variability not related to the study hypothesis. First, years of education were included as a proxy of cognitive reserve, as carried out in recent clinical and epidemiological research [49,50]. In alignment with a bi-componential framework of reserve [51] and with a dual view of active and passive processes contributing to inter-individual variability in the association between retained neural resources and behavioural functioning [52], two MRI-derived indices were also added to the models as covariates: brain parenchymal ratio and left hippocampal volume (see Section 2.2.2 for methodological details on how these values were obtained). The former was included as a dynamic index of brain reserve [53], whereas the latter served as a proxy of neurodegeneration typically associated with AD (i.e., a left-lateralised ROI was chosen, given the language-based nature of the BNT). Age, finally, was added as a fourth covariate.

Model 2 tested the statistical effect of qualitative scoring (i.e., the average SMI score of correctly named BNT items). This model was corrected for the four aforementioned covariates and, additionally, for quantitative scores that were included as a fifth covariate. This served to identify the statistically independent effect of qualitative from quantitative scoring. In Model 2, we tested the negative association between qualitative scores and GM, based on the principle whereby words with a lower SMI score tend to be considered more difficult.

Given the exploratory nature of the analyses, both Model 1 and Model 2 were thresholded at a cluster-forming threshold of *p* < 0.01, and corrected for Family-Wise Error (FWE) at a cluster level. No further corrections for multiple comparisons were applied. Only clusters surviving an FWE-corrected *p* < 0.05 were reported as significant. To verify maps of structural covariance of the left hippocampus, two multiple-regression models (one for each sub-cohort) were launched, using ROI volumes as a predictor of global GM maps. These analyses were corrected for age, intracranial volume, and GM ratio, and were thresholded at a complete FWE-corrected *p* < 0.05. Peak coordinates were converted from the Montreal Neurological Institute space to the Talairach space via a nonlinear transform and were interpreted using the Talairach Daemon client (www.talairach.org/daemon.html).

## 3. Results

### 3.1. Descriptive Variables

Main demographic descriptors and other variables relevant to this study are summarised in Table 1. On average, MRI data were acquired at a temporal distance of approximately 22 days from the administration of the BNT (i.e., maximum absolute distance: 83 and 92 days, in control and MCI individuals, respectively). Although the two sub-cohorts were analysed separately, controls were slightly older and had significantly more balanced (i.e., ~50%/50%) proportions of males and females. As expected, a larger proportion of APOE ɛ_4_ carriers was observed among MCI individuals. The latter also scored significantly lower on the Mini Mental State Examination and on the quantitative BNT performance (calculated on either all 30 items or only on those 27 with a valid SMI score). The proportion of controls and MCI individuals who named each of the 27 BNT items correctly (and the statistical differences between the two diagnostic groups) is reported in Table 2. As expected, words with a higher SMI score tended to be named more often, as shown by a positive coefficient of correlation between SMI scores and the proportion of participants who correctly named each item (controls: *rho*_27_ = 0.455; MCI participants: *rho*_27_ = 0.488).

### 3.2. Model 1: Quantitative BNT Performance

**Figure 1:**
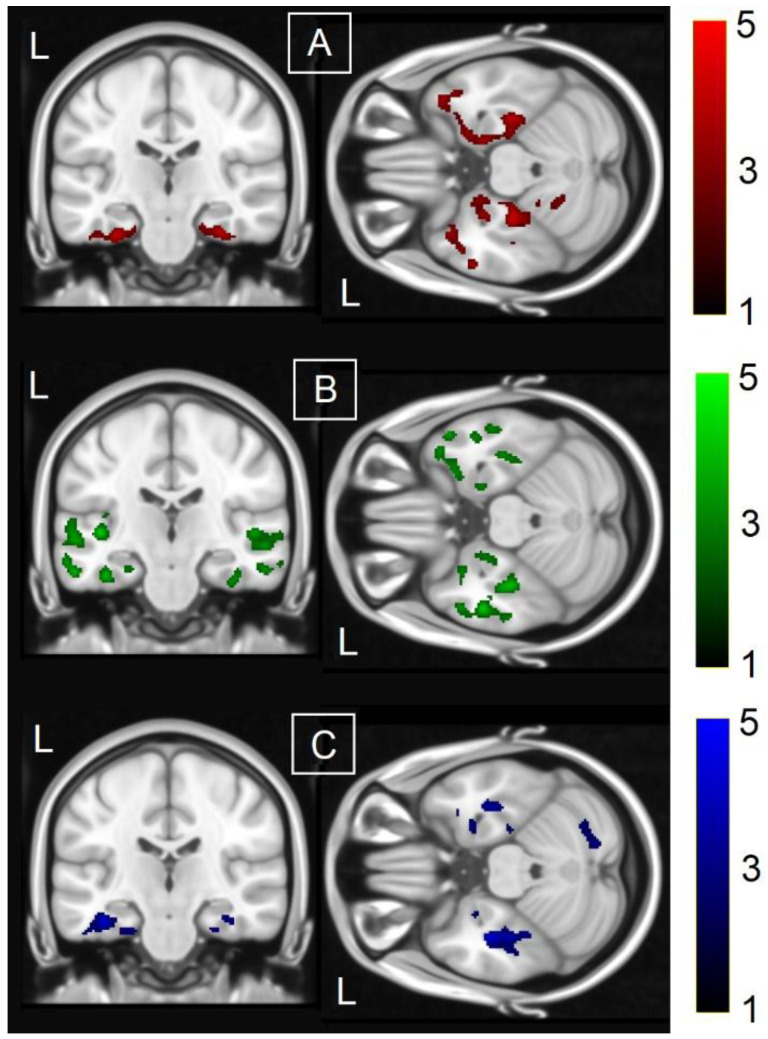
Voxel-based analyses showing a significantly association between GM and: (**A**) quantitative BNT performance in the sub-cohort of cognitively healthy controls (positive—red); (**B**) quantitative BNT performance in the MCI sub-cohort (positive—green); (**C**) qualitative BNT performance in the MCI sub-cohort (negative—blue). *z*-Scores are illustrated with the coloured scale on the right hand-side. Analyses were adjusted for age, years of education, brain parenchymal ratio, and left hippocampal volume. Statistical parametric maps were thresholded at FWE-corrected *p* < 0.05 and rendered on the Montreal Neurological Institute 152 T1-weighted template (shown slices are y = −22 and z = −26).

In the sub-cohort of controls, a significant positive association was found between quantitative BNT performance and GM density in three large clusters centred around the temporal and mediotemporal lobe, bilaterally, and extending to part of the prefrontal, cerebellar, and striatal territory (Table 3, Figure 1A).

In the sub-cohort of individuals with a diagnosis of MCI, a significant positive association was found between quantitative BNT performance and GM density in three clusters (Table 4). Two of these were located in the temporal and mediotemporal lobe, bilaterally, and a third one stretched to the territory of the anterior right insula. When compared with the clusters found in the sub-cohort of controls, these results covered a larger portion of the temporal lobe, expanding to the superior temporal cortex and middle temporal gyrus (Figure 1B).

### 3.3. Model 2: Qualitative BNT Performance

No significant results were found in the sub-cohort of cognitively healthy controls.

In the sub-cohort of individuals with MCI, a significant negative association was found between average SMI and GM density in two ventromedial clusters located bilaterally in the temporal lobe (Table 5; Figure 1C). These included the anterior fusiform gyrus (BA20), the entorhinal cortex (BA28), and the perirhinal cortex (BA35), and extended to the right posterolateral cerebellum (Crus II).

No significant cluster was found in association with somatosensory or motor areas.

### 3.4. Post Hoc ROI Analyses

To complement voxel-based models, ROI analyses were run with a specific focus on the mediotemporal regions that are typically linked to SM retrieval [9]. The same methodology described in Section 2.2.2 in relation to the left hippocampus was adopted to extract regional volumes also from the right hippocampus, the left and right entorhinal cortices (Brodmann Area 28), and the left and right perirhinal cortices (Brodmann Area 35). ROI validity was scrutinised via structural covariance models (as those described in Section 2.2.2 in relation to the left hippocampus). The outcome maps confirmed the validity of the extracted scores (see Supplementary Figure S1 for an illustration of these resulting maps).

**Figure 2:**
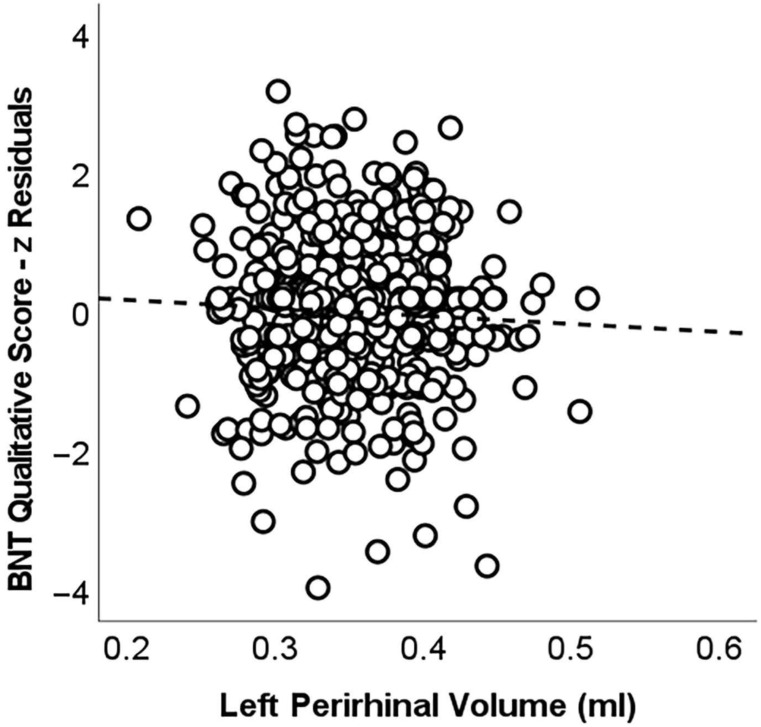
Scatterplot illustrating the association between volumes of the left perirhinal cortex and qualitative BNT scores in the MCI sub-cohort. To capture this association, after accounting for quantitative BNT scores, residual scores were calculated. The line represents the *β* coefficient calculated based on the coefficient of correlation (*r*_350_ = −0.089; *β* = −0.004).

To test for the independent association between mediotemporal volumes and qualitative BNT scores, standardised residual scores were calculated to regress out quantitative BNT performance from qualitative scores. Coefficients of correlation were then run to test the association between the residual scores and volumes of the aforementioned selected brain regions. Of all one-tailed *p*-values (significance was tested in a single direction to reflect the outcome of whole-brain analyses), only a single *p*-value was significant. There was a modest but significant correlation between the BNT residual qualitative score and the volume of the left perirhinal cortex in the MCI sub-cohort (*r_350_* −0.089, *p* = 0.048; Figure 2).

## 4. Discussion

We studied the association between GM structural integrity and the quantitative and qualitative aspects of BNT performance, scored via an item-level method. To operationalise qualitative aspects, we focused on the SMI score of each word in order to explore semantic difficulty in association with a well-defined thematic aspect (linked to pericentral somatosensory and motor regions). The findings indicate that item-level scores predict an independent portion of GM variability in the temporal and mediotemporal lobe of individuals with MCI, with a major peak of significance emerging from the perirhinal portion of the anterior parahippocampal gyrus. This supports our first hypothesis, as qualitative BNT scoring expands the characterisation of semantic processing beyond “traditional” BNT scores. However, our second hypothesis is not supported by the findings, as no significant association was found in the proximity of somatosensory or motor areas. Qualitative scores were also associated with GM density in the right posterolateral cerebellum. This cerebellar region (corresponding to Crus II) shows functional connectivity with the cerebral hubs of the default-mode network [54] and is involved in semantic processing [55].

Successful naming of BNT items depends on a set of cognitive abilities, including sensorimotor functioning (i.e., visual perception and phono-articulatory skills), visual recognition, SM, and lexical access [56]. Although it is not possible to link incorrectly named items to deficits or setbacks in any specific function from the above list, people who are neurologically healthy and individuals diagnosed with MCI typically do not have any sensorimotor difficulties. Moreover, the sense of familiarity elicited by BNT images is typically very high among older adults, with scores ranging between 7.00 and 6.37 (the latter was in association with “ABACUS”, i.e., the 60th and, presumably, the hardest BNT item) on a 1 to 7 Likert scale (with 1 being “not at all familiar” and 7 being “very familiar”) [57]. Comparably, an analysis of familiarity elicited in an anterograde visual recognition task showed that this very basic sub-process of declarative memory is preserved in MCI [58]. This indicates that incorrect BNT trials are due to lexical/SM access in these two populations. Although qualitative and quantitative scores were strongly correlated in the sub-cohort of MCI individuals (*r*_350_ = −0.720), the variance inflation factor between the two variables was within the acceptable range (*VIF* = 2.078), and qualitative scores predicted an independent portion of GM density in the temporal and mediotemporal lobe, i.e., a region that plays a central role in SM. This, however, only emerged as a modest correlation when data were analysed via an ROI-based approach, indicating that, although qualitative BNT performance does provide additional evidence about the integrity of regions that sustain lexical–semantic access beyond quantitative scores, this added value is limited. The limited number of items included in the ADNI version of the BNT (i.e., *n* = 30) leads to ceiling effects more easily than the original 60-item version of the test, and this cluster of datapoints contributes to the width of the correlation between quantitative and qualitative scores (i.e., we found that 50 controls and 42 MCI participants obtained a flawless 27 out of 27 quantitative score, and this resulted in an average SMI of 4.931 for all of them). We argue that, with the inclusion of more BNT items and the subsequent increase in numerical variability, the relation between quantitative and qualitative performance may become weaker, similarly to what is observed with the Category Fluency Test.

In a study carried out in a sample of 76 early-AD participants, the standard, quantitative performance on the abbreviated 15-item BNT included as part of the Consortium to Establish a Registry for Alzheimer’s Disease was associated with a standardised uptake ratio of the radiolabelled Pittsburgh Compound B tracer (i.e., sensitive to amyloid pathology) in the left entorhinal cortex [59]. A second neuromolecular study was carried out in a sample of 64 participants with AD of variable severity (mild to severe) and used AV1451 (also known as “Flortaucipir”), a radiotracer that selectively binds to neurofibrillary tangles of hyperphosphorylated TAU. BNT performance was not associated with tracer uptake in the mediotemporal lobe in this cohort, but was associated with a widespread pattern that extended to the anterior cingulate and to the frontal, temporal, and parietal lobes [60]. This finding indicates that quantitative BNT scores might be sensitive to underlying AD-related changes in the neural tissue of the mediotemporal lobe, but this might be visible only during the earliest phases of the disease. In this respect, the pattern of mediotemporal GM associations we found in both sub-cohorts (Figure 1A,B) might be partly linked to variability in underlying AD mechanisms. No study, to our knowledge, has investigated the link between the neuromolecular imaging of AD pathology and BNT in healthy controls. A study carried out in a mixed sample (*n* = 96) of healthy older controls and participants with AD who underwent lumbar puncture, however, found that a lower amyloid/TAU ratio is a significant predictor of BNT performance [61]. This indicates that more studies are needed to characterise the link between quantitative BNT performance and early AD pathology. In light of the findings of this study, we argue that the additional inclusion of qualitative BNT scores may allow for a more fine-grained characterisation of neuroimaging-informed patterns indicative of amyloid and TAU pathology.

Although BNT items are roughly sorted in order of difficulty (in this study, from Item 1: “BED” to Item 59: “PROTRACTOR”), item-level scores assessing words’ “frequency of use” indicate that the difficulty levels of individual items fluctuate throughout task administration [62]. This legitimises a more in-depth focus on individual items, as different items are associated with different semantic difficulties, and two performances that are quantitatively equal may be qualitatively different. Interestingly, however, our second hypothesis is not supported by the results, as we did not find any significant cluster/coordinate that extended to the somatosensory or motor area. The main reason we selected SMI as an index of semantic difficulty was to be able to visualise its independent effects, as these may have been visible in a topographically different set of regions. SMI facilitates semantic processing (as referents with higher potential for interaction are associated with a stronger semantic activation [63]) and is also linked to a better verbal free recall performance [64]. The findings of the current study, however, suggest that SMI ended up being a *general* index of semantic difficulty, rather than a *specific* feature capturing potential for body–object interactions. Arguably, a larger set of BNT items will result in larger score variability, and this, in turn, may allow SMI to express its construct validity fully.

The visual comparison of the findings obtained in the MCI sub-cohort suggests that qualitative scores may be less influenced by semantic control processes than quantitative scores. The unique portion of GM variability that accounted for qualitative scores, in fact, was limited to temporal, mediotemporal, and cerebellar default-mode-related areas, whereas quantitative scores were associated with a much wider pattern that also extended to other cortical regions. Although it is not possible to interpret naming difficulties (i.e., resulting in variability in quantitative scores) in terms of failure in access/control processes, standard quantitative scoring gives the participant a second and third chance by providing them with semantic and phonological cues that help direct semantic control resources [65]. Vice versa, the variability of qualitative scores is captured as an average of correctly named items and should thus be less susceptible to the influence of semantic control. This suggests that the use of qualitative scores could flank the standard quantitative scores in constructing the profile of lexical–semantic access (and, more generally, declarative memory) of individuals who undergo neuropsychological testing.

### Limitations

As this study was, to our knowledge, the first one to explore the item-level scoring of BNT performance, we gave particular emphasis to methodological and feasibility-related aspects, but did not equally investigate the clinical value of the approach (e.g., by focusing on diagnostic differences). We expect that this methodology may be effective, for instance, at detecting abnormal lexical–semantic processing in tertiary-care neurological settings or in samples of community-dwelling adults. This is an area that will be worth investigating with *ad hoc* research projects. Along similar lines, we relied on ADNI’s clinical diagnoses but did not include biomarker status in our design. This investigation was mostly concerned about the effectiveness of the methodology, but we recognise that the inclusion of biomarkers (e.g., to test the association between item-level lexical–semantic indices and peripheral levels of pathology) could help establish the effectiveness of this method at the level of the individual clinical referral. A third clinical aspect to consider is longitudinal follow ups and the extent to which this methodology can highlight AD progression and/or is influenced by test–retest practice effects. Finally, applications in the context of other neurological conditions (e.g., semantic dementia), in relation to other axes of semantic difficulty (e.g., ‘age of acquisition’ or ‘frequency of use’) or, more generally, the transposition of item-level methodological principles to the scoring of other verbal tests (e.g., the Prose Memory Test [66]) are also warranted.

## 5. Conclusions

The aim of this research was to assess the value of item-level scoring of BNT performance in relation to “standard” quantitative scoring. Whereas quantitative BNT scores are routinely used as a measure of lexical–semantic access, qualitative scores may provide a more fine-grained proxy of SM that is less influenced by semantic control. To test this idea, we designed two voxel-based models assessing the linear association between GM density and quantitative and SMI-informed qualitative BNT scores. We found that the item-level scoring of BNT performance is a significant predictor of temporal and mediotemporal GM in people with MCI. Albeit modest, this statistical effect is independent of traditional quantitative scores that are based on an item count. This may inform the design of *ad hoc* studies that could help evaluate the clinical potential of this approach.

It is important to design alternative methodologies to maximise the informativity of neuropsychological tests. This is particularly relevant for methodologies that can be applied retrospectively, not by collecting new data, but by devising novel opportunities based on innovative post-processing approaches. Item-level scores obtained from verbal tests may be less susceptible to semantic control mechanisms or to non-semantic sources of variability. This is crucial to define cognitive indices that are sensitive to parahippocampal damage that is often silent during the preclinical stage of AD.

## Figures and Tables

**Table 1:** Demographic characterisation of the two sub-cohorts.

Descriptor	Controls	MCI	Test Statistic	*p*-Value
Age (years)	76.07 (4.93)	74.90 (7.24)	*t_df=_*_525.941_ = 2.233	0.026
Education (years)	16.14 (2.93)	15.76 (2.91)	*t_df=_*_545_ = 1.459	0.145
Gender (F/M)	97/100	118/232	*χ^2^_df=_*_1_ = 12.734	<0.001
Handedness (L/R)	13/184	27/322 *	*χ^2^_df_*_=1_ = 0.240	0.624
APOE genotype (ε_2_ε_2_/ε_2_ε_3_/ε_3_ε_3_/ε_4_ε_2_/ε_4_ε_3_/ε_4_ε_4_)	2/27/120/1/43/4	0/15/141/9/142/43	*χ^2^_df_*_=5_ = 60.822	<0.001
MRI scan—BNT test distance (days)	20.89 (13.58)	22.57 (14.39)	*t_df=_*_545_ = −1.338	0.182
Mini Mental State Examination	29.15 (0.94)	27.04 (1.80)	*t_df=_*_542.435_ = 18.030	<0.001
*N* of correctly named BNT trials (*n_max_* = 30)	27.43 (2.68)	25.24 (3.99)	*t_df=_*_528.282_ = 7.632	<0.001
*N* of correctly named BNT trials (*n_max_* = 27)	24.83 (2.29)	22.91 (3.54)	*t_df=_*_534.584_ = 7.658	<0.001

* Handedness information was not available for one of the participants with MCI. APOE: Apolipoprotein E; BNT: Boston Naming Test; F: female; M: male; MCI: mild cognitive impairment.

**Table 2:** SMI normative scores and naming frequency per BNT item.

BNT Item	Word	SMI	Correct Naming (Proportion)		*p* *(* *χ^2^)*
			Controls (*n* = 197)	MCI (*n* = 350)	
01	“BED”	6.360	1.000	1.000	N/A
03	“PENCIL”	5.870	1.000	1.000	N/A
05	“WHISTLE”	4.478	1.000	0.989	0.132
07	“COMB”	5.923	1.000	1.000	N/A
09	“SAW”	4.760	1.000	0.994	0.288
11	“HELICOPTER”	5.179	0.990	0.969	0.117
15	“HANGER”	5.292	1.000	1.000	N/A
17	“CAMEL”	4.522	1.000	0.986	0.092
19	“PRETZEL”	6.040	0.959	0.826	<0.001
21	“RACQUET”	5.240	0.990	0.974	0.213
25	“DART”	5.292	0.964	0.914	0.025
27	“GLOBE”	5.280	0.975	0.906	0.002
29	“BEAVER”	3.826	0.827	0.711	0.003
31	“RHINOCEROS”	3.680	0.924	0.814	<0.001
33	“IGLOO”	4.852	0.980	0.940	0.033
35	“DOMINOES”	5.704	0.919	0.851	0.022
37	“ESCALATOR”	5.520	0.975	0.911	0.004
39	“HAMMOCK”	6.167	0.964	0.886	0.002
41	“PELICAN”	3.667	0.843	0.720	0.001
43	“PYRAMID”	4.000	0.934	0.857	0.007
45	“UNICORN”	1.296	0.807	0.603	<0.001
47	“ACCORDION”	5.826	0.934	0.823	<0.001
49	“ASPARAGUS”	5.560	0.985	0.906	<0.001
51	“LATCH”	5.542	0.624	0.591	0.450
53	“SCROLL”	4.227	0.909	0.800	<0.001
55	“SPHINX”	3.120	0.802	0.603	<0.001
59	“PROTRACTOR”	5.435	0.523	0.340	<0.001

“RACQUET” was scored based on the American English spelling “RACKET”, as reported by SMI norms [36]. The difference between “RHINO” (SMI = 3.875) and “RHINOCEROS” (SMI = 3.680) was negligible, and the latter score was used. Items 13 (“OCTOPUS”), 23 (“VOLCANO”), and 57 (“TRELLIS”) were not included in the analyses, as they missed a normative SMI score. BNT: Boston Naming Test; MCI: mild cognitive impairment; N/A: not assessed (no difference between the two sub-cohorts); SMI: sensorimotor interaction.

**Table 3:** Association between GM density and quantitative BNT scores in the sub-cohort of cognitively healthy controls.

Cluster Number	Cluster Level *p (FWE)*	Cluster Extent (Voxels)	Side	Brodmann Area	Brain Region	*z*-Score at Local Maximum	Talairach Coordinate		
							x	y	z
1	<0.001	5250	L	20	Uncus	4.74	−33	−16	−29
				28	Uncus	4.73	−28	−11	−32
				47	Inferior Frontal Gyrus	4.24	−24	14	−18
				20	Uncus	4.12	−28	0	−37
				20	Fusiform Gyrus	4.11	−45	−34	−16
				36	Parahippocampal Gyrus	3.98	−30	−24	−21
					Hippocampus	3.90	−27	−13	−18
					Cerebellum-Culmen	3.76	−28	−57	−26
				47	Inferior Frontal Gyrus	3.71	−32	22	−18
				38	Superior Temporal Gyrus	3.71	−33	1	−14
				21	Middle Temporal Gyrus	3.69	−56	−4	−20
				20	Inferior Temporal Gyrus	3.61	−50	−16	−28
				38	Superior Temporal Gyrus	3.60	−24	6	−33
				37	Inferior Temporal Gyrus	3.53	−44	−44	−15
2	0.006	1281	L		Cerebellar Tonsil	4.50	−20	−49	−45
3	<0.001	4418	R		Putamen	4.41	30	3	7
				30	Limbic Sub-Gyral	4.34	16	−41	−5
				36	Parahippocampal Gyrus	4.29	30	−33	−16
				20	Uncus	4.13	32	−13	−28
				20	Uncus	4.10	30	−9	−33
				19	Lingual Gyrus	3.97	15	−47	−1
				36	Parahippocampal Gyrus	3.88	32	−22	−22
				38	Superior Temporal Gyrus	3.82	33	12	−34
				20	Inferior Temporal Gyrus	3.71	44	−4	−38
				21	Middle Temporal Gyrus	3.71	45	1	−35
				38	Superior Temporal Gyrus	3.64	44	14	−21
				28	Parahippocampal Gyrus	3.60	20	−11	−22
				38	Superior Temporal Gyrus	3.56	53	9	−9
				38	Superior Temporal Gyrus	3.52	34	14	−33

Only *z*-scores > 3.50 at local maximum are reported. FWE: Family-Wise Error; L: left; R: right.

**Table 4:** Association between GM density and quantitative BNT scores in the sub-cohort of MCI participants.

Cluster Number	Cluster-Level *p (FWE)*	Cluster Extent (Voxels)	Side	Brodmann Area	Brain Region	*z*-Score at Local Maximum	Talairach Coordinate		
							x	y	z
1	<0.001	5881	L	20	Inferior Temporal Gyrus	5.01	−51	−9	−23
				21	Temporal Sub-Gyral	4.42	−42	−5	−13
					Insula	4.31	−44	−20	1
				21	Middle Temporal Gyrus	4.20	−50	−3	−12
				20	Uncus	4.18	−28	0	−35
				21	Middle Temporal Gyrus	3.97	−56	−9	−11
				20	Inferior Temporal Gyrus	3.94	−56	−24	−17
				22	Superior Temporal Gyrus	3.93	−51	−29	4
				20	Fusiform Gyrus	3.83	−38	−26	−21
				22	Middle Temporal Gyrus	3.78	−56	−32	5
				21	Superior Temporal Gyrus	3.75	−56	−23	−1
				21	Middle Temporal Gyrus	3.73	−61	−39	−1
				21	Middle Temporal Gyrus	3.67	−45	1	−29
				38	Superior Temporal Gyrus	3.63	−30	4	−27
2	0.002	1635	R	13	Insula	4.81	36	8	13
					Claustrum	4.09	26	20	2
				44	Precentral Gyrus	3.69	42	16	7
3	<0.001	4595	R	38	Superior Temporal Gyrus	4.30	40	−1	−15
				38	Middle Temporal Gyrus	3.98	39	7	−37
				20	Inferior Temporal Gyrus	3.95	44	1	−38
				20	Uncus	3.95	33	−9	−31
				22	Superior Temporal Gyrus	3.92	57	−2	−2
				21	Middle Temporal Gyrus	3.73	50	−12	−11
				20	Fusiform Gyrus	3.73	50	−3	−23
				21	Middle Temporal Gyrus	3.66	61	−9	−6
				38	Superior Temporal Gyrus	3.53	53	11	−21

Only *z*-scores > 3.50 at local maximum are reported. FWE: Family-Wise Error; L: left; R: right.

**Table 5:** Association between GM density and qualitative BNT scores in the sub-cohort of MCI participants.

Cluster Number	Cluster-Level *p (FWE)*	Cluster Extent (Voxels)	Side	Brodmann Area	Brain Region	*z*-Score at Local Maximum	Talairach Coordinate		
							x	y	z
1	0.004	1434	L	20	Fusiform Gyrus	4.27	−39	−26	−17
				28	Uncus	4.26	−26	−9	−27
				20	Temporal Sub-Gyral	4.11	−39	−15	−22
				35	Parahippocampal Gyrus	4.00	−22	−16	−26
2	0.001	1650	R	36	Parahippocampal Gyrus	3.71	36	−30	−16
				20	Uncus	3.62	27	−5	−35
					Claustrum	3.53	38	−2	8
3	<0.001	2370	R	19	Lingual Gyrus	3.53	20	−68	−4

Only *z*-scores > 3.50 at local maximum are reported. FWE: Family-Wise Error; L: Left; R: Right.

## Data Availability

All data are publicly available at https://adni.loni.usc.edu/.

## Supplementary Materials

The following supporting information can be downloaded at https://www.mdpi.com/article/10.3390/brainsci13050806/s1: Figure S1: Structural covariance maps of mediotemporal regions of interest (ROIs).
