# Supplementary material for: Item-Level Scores on the Boston Naming Test as an Independent Predictor of Perirhinal Volume in Individuals with Mild Cognitive Impairment
Source: Brain Sci. 2023 May 16;13(5):806. doi: 10.3390/brainsci13050806 (PMC10216160; doi:10.3390/brainsci13050806)
Supplement: Supplementary file 1 [file brainsci-13-00806-s001.zip › brainsci-2363466-supplementary.pdf]

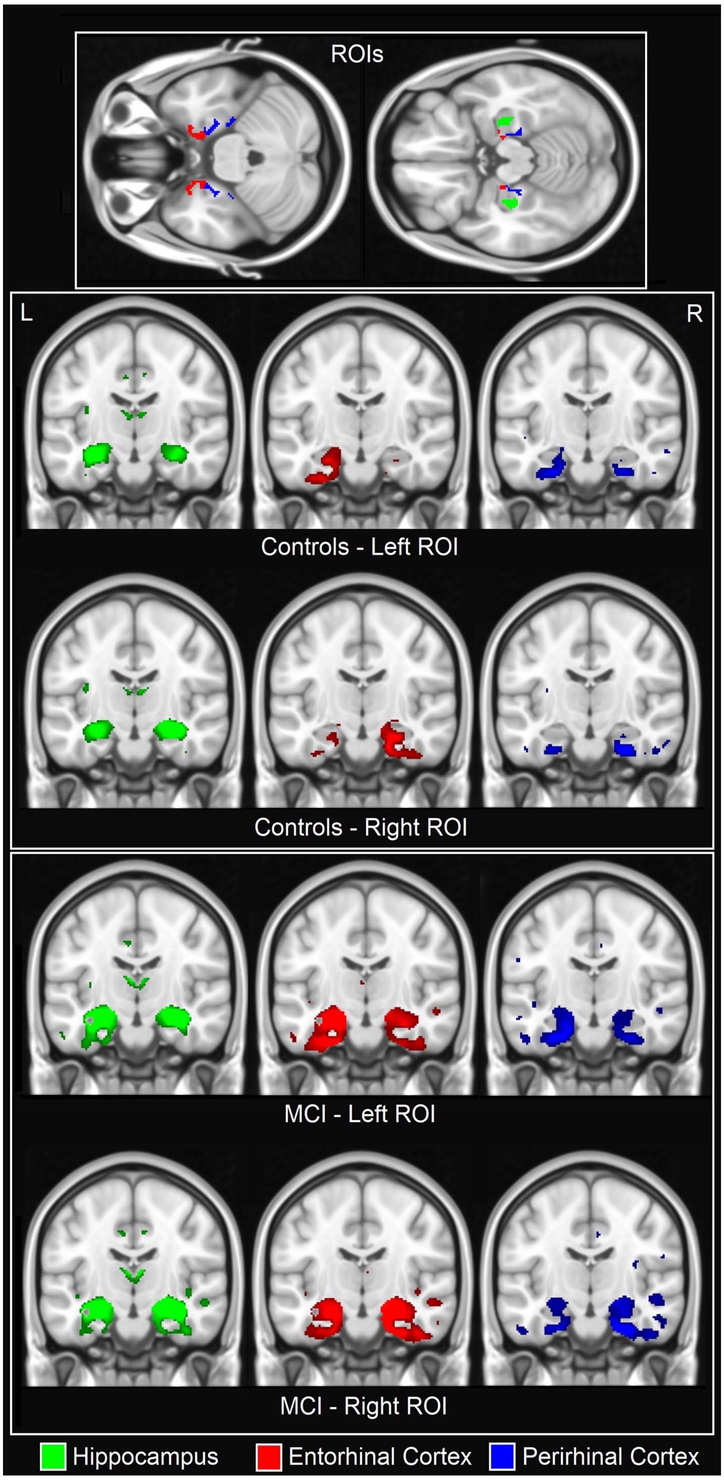

Structural covariance maps of mediotemporal regions of interest (ROIs): hippocampus, entorhinal cortex and perirhinal cortex. The three ROIs are shown in the top row of this figure. Maps were calculated separately for each diagnostic sub-cohort and for each hemisphere. MCI: mild cognitive impairment.
